# A Circulating MicroRNA Profile Is Associated with Late-Stage Neovascular Age-Related Macular Degeneration

**DOI:** 10.1371/journal.pone.0107461

**Published:** 2014-09-09

**Authors:** Felix Grassmann, Peter G. A. Schoenberger, Caroline Brandl, Tina Schick, Daniele Hasler, Gunter Meister, Monika Fleckenstein, Moritz Lindner, Horst Helbig, Sascha Fauser, Bernhard H. F. Weber

**Affiliations:** 1 Institute of Human Genetics, University of Regensburg, Regensburg, Germany; 2 Department of Ophthalmology, University Hospital Regensburg, Regensburg, Germany; 3 Department of Ophthalmology, University Hospital of Cologne, Cologne, Germany; 4 Biochemistry Center Regensburg (BZR), Laboratory for RNA Biology, University of Regensburg, Regensburg, Germany; 5 Department of Ophthalmology, University of Bonn, Bonn, Germany; Copenhagen University Hospital Roskilde and the University of Copenhagen, Denmark

## Abstract

Age-related macular degeneration (AMD) is the leading cause of severe vision impairment in Western populations over 55 years. A growing number of gene variants have been identified which are strongly associated with an altered risk to develop AMD. Nevertheless, gene-based biomarkers which could be dysregulated at defined stages of AMD may point toward key processes in disease mechanism and thus may support efforts to design novel treatment regimens for this blinding disorder. Circulating microRNAs (cmiRNAs) which are carried by nanosized exosomes or microvesicles in blood plasma or serum, have been recognized as valuable indicators for various age-related diseases. We therefore aimed to elucidate the role of cmiRNAs in AMD by genome-wide miRNA expression profiling and replication analyses in 147 controls and 129 neovascular AMD patients. We identified three microRNAs differentially secreted in neovascular (NV) AMD (hsa-mir-301-3p, p_corrected_ = 5.6*10^−5^, hsa-mir-361-5p, p_corrected_ = 8.0*10^−4^ and hsa-mir-424-5p, p_corrected_ = 9.6*10^−3^). A combined profile of the three miRNAs revealed an area under the curve (AUC) value of 0.727 and was highly associated with NV AMD (p = 1.2*10^−8^). To evaluate subtype-specificity, an additional 59 AMD cases with pure unilateral or bilateral geographic atrophy (GA) were analyzed for microRNAs hsa-mir-301-3p, hsa-mir-361-5p, and hsa-mir-424-5p. While we found no significant differences between GA AMD and controls neither individually nor for a combined microRNAs profile, hsa-mir-424-5p levels remained significantly higher in GA AMD when compared to NV (p_corrected_<0.005). Pathway enrichment analysis on genes predicted to be regulated by microRNAs hsa-mir-301-3p, hsa-mir-361-5p, and hsa-mir-424-5p, suggests canonical TGFβ, mTOR and related pathways to be involved in NV AMD. In addition, knockdown of hsa-mir-361-5p resulted in increased neovascularization in an *in* *vitro* angiogenesis assay.

## Introduction

Age-related macular degeneration (AMD) is a highly prevalent cause of severe vision impairment among people aged 55 years and older [1]. It is a degenerative disorder of the central retina involving predominantly the rod photoreceptors, the retinal pigment epithelium (RPE), Bruchs membrane and the underlying choriocapillaris [2]. The disease aetiology is complex and is influenced by a combination of multiple genetic susceptibility factors and environmental components.

An early sign of AMD is the appearance of drusen, yellowish extracellular deposits of protein and lipid material within and beneath the RPE. Advanced AMD manifests essentially as two distinct late-stage lesions – geographic atrophy (GA) and neovascular (NV) AMD. GA occurs in up to 50% of cases and is clinically defined as a discrete area of RPE atrophy with visible choroidal vessels in the absence of neovascularization in the same eye [2]–[5]. It may or may not involve the fovea. NV AMD describes the development of new blood vessels beneath and within the retina and is characterized by serous or hemorrhagic detachment of either the RPE or the sensory retina, the presence of subretinal fibrous tissue and eventually widespread RPE atrophy. Progression to visual loss can be rapid in NV AMD [1].

The precise aetiology of AMD is still not fully understood, although risk factors such as age, smoking, and genetic components are known to strongly contribute to disease development [2]. In Western societies, AMD reveals an age-dependent prevalence of almost 1 in 5 people aged 85 and above [3]–[5]. Across a number of epidemiological studies, smoking has consistently been associated with increased risk of developing advanced AMD with an estimated odds ratio of approximately 2 [6]. The exact mechanism, however, by which smoking affects the retina is unknown. Twin studies and familial aggregation studies suggested a significant genetic contribution of up to 70% in disease risk [7]. Subsequently, several genes have been implicated in AMD pathology by candidate gene studies as well as genome wide association studies. Genetic variants in complement factor H (CFH) and ARMS2/HtrA Serine Protease 1 (HTRA1) were found to be strongly associated with odds ratios over 2.5 per risk allele. In addition, multiple medium to low effect size gene variants were discovered in a large number of loci across the genome. A recent meta-analysis of genome wide association studies found a total of 19 independently associated loci by comparing over 17,000 cases and 60,000 controls [8].

The combined effect of the major risk variants on AMD was estimated by modelling risk scores [9]. The multiple logistic regression model was found to have an area under the curve (AUC) of about 82%, which is suitable for classifying individuals in high and low risk groups. Accordingly, roughly 50% of AMD cases and 50% of healthy controls can now reliably be predicted. However, a large proportion of AMD cases do not have the expected genetic risk profile despite their given disease status. Consequently, other components, genetic or environmental, may influence disease development. This makes it crucial to identify these components possibly by defining disease biomarkers correlating with the underlying genetic or environmental factors and eventually reflecting a defined disease stage.

Recently, circulating microRNAs (cmiRNAs) were found in blood plasma or blood serum where they are carried by nanosized exosomes or microvesicles [10], [11]. Origin and effects of these cmiRNAs are unclear although some studies suggested functional involvement in cell-to-cell signalling [12]. In general, cmiRNAs are potential biomarkers which can be used for diagnostics and prognostics of human diseases [13]. Additionally, synthetic microRNAs in artificial exsosomes could be applicable for therapeutic approaches by modulating cmiRNA levels.

In this study, we aimed to elucidate the role of cmiRNAs in AMD and performed a genome-wide expression profiling in patients affected by late stage neovascular manifestation. Such analyses provide a promising approach to define biomarkers for AMD which could be helpful to identify as of yet unknown gene targets involved in defined aspects of AMD pathology. Such biomarkers could also serve as the long sought-after variable needed to monitor treatment effects in future clinical trials for AMD.

## Results

### Study design

We applied a three stage design to identify significantly associated cmiRNAs. First, RNASeq was performed to screen for miRNA candidates in 9 cases and 9 controls from the Regensburg study. The cmiRNAs with a nominal significance of p>0.1 were then validated in an unrelated set of 45 NV cases and 68 controls from the Regensburg study (**Table 1**). Finally, candidate cmiRNAs with a nominal significant association (p<0.05, adjusted or unadjusted for glaucoma) and an odds ratio above 2 or below 0.5 were then replicated in a population based study (Cologne study, **Table 1**) consisting of 75 NV cases and 70 controls. In total, the combined study included 129 patients with NV AMD and 147 AMD-free controls (**Table 1**). Additionally, 59 AMD patients with pure GA were assessed for candidate cmiRNAs to test for specificity of the findings in NV AMD.

**Table 1:** Summary characteristics of the study.

	Regensburg	Bonn	Cologne
Study type	Case/Control	Case/Control	Population based
Number of individuals	131	18	186
Controls	77	0	70
Cases	54	18	116
Geographic atrophy	0	18	41
Neovascular AMD	54	0	75
Mean age cases (S.D.) [years]	75.15 (6.75)	74.60 (8.70)	80.22 (9.24)
Mean age controls (S.D.) [years]	73.26 (8.00)	-	78.44 (8.76)
Female cases [%]	59.3	61.1	56.9
Female controls [%]	54.5	-	55.7
Glaucoma in cases [%]	11.1	5.5	NA
Glaucoma in controls [%]	83.1	-	NA

### Identification of cmiRNAs in NV AMD (discovery study)

To search for candidate cmiRNAs, we first performed next-generation sequencing of cmiRNAs extracted from plasma of 9 AMD NV cases and 9 matched controls. Overall, in the 18 samples we identified 203 different cmiRNA species. Of these, 10 cmiRNAs were significantly associated with late-stage NV AMD (p_uncorrected_<0.1) (**Table 2**).

**Table 2:** Association of circulating microRNAs with AMD in the Regensburg discovery study (9 NV cases and 9 controls).

microRNA	uncorrected p-value	mean cases (95% CI)	mean controls (95% CI)
hsa-miR-142-5p	0.012	1.21 (1.14–1.28)	1.00 (0.93–1.07)
hsa-miR-192-5p	0.010	1.29 (1.20–1.38)	1.00 (0.91–1.09)
hsa-miR-194-5p	0.028	1.28 (1.19–1.38)	1.00 (0.89–1.11)
hsa-miR-26a-5p	0.082	0.90 (0.83–0.96)	1.00 (0.94–1.06)
hsa-miR-301a-3p	0.084	0.83 (0.72–0.93)	1.00 (0.90–1.10)
hsa-miR-335-5p	0.094	1.34 (1.17–1.50)	1.00 (0.84–1.16)
hsa-miR-361-5p	0.056	0.74 (0.56–0.91)	1.00 (0.85–1.15)
hsa-miR-424-5p	0.028	0.52 (0.30–0.73)	1.00 (0.84–1.16)
hsa-miR-4732-5p	0.086	1.24 (1.12–1.36)	1.00 (0.88–1.12)
hsa-miR-505-5p	0.048	1.29 (1.13–1.44)	1.00 (0.85–1.15)

### Circulating miRNAs associated with NV AMD (replication study)

To replicate the initial findings, qRT-PCR was performed for the significant 10 cmiRNAs in 113 samples consisting of 45 NV AMD cases and 68 controls. Three cmiRNAs were identified (hsa-mir-301-3p, hsa-mir-361-5p, and hsa-mir-451a-5p) which showed (1) an association signal in the same direction as in the discovery study, (2) an odds ratio over 2 or under 0.5 and (3) an uncorrected (one-sided) p-value below 0.1. These three cmiRNAs showed reduced levels in the serum of CNV cases compared to AMD free controls. The association was robust also when adjusting for covariates such as age, gender, smoking (measured in packyears), genetic risk score (GRS) or levels of the housekeeping cmiRNA hsa-mir-451a-5p (**Table 3**). Of note, two cmiRNAs (hsa-mir-301-3p and hsa-mir-361-5p) were strongly confounded by glaucoma disease status and showed stronger association signals when adjusting for glaucoma.

**Table 3:** Sensitivity analysis in the Regensburg study by multiple logistic regression models.

covariate	hsa-miR-301a-3p	hsa-miR-361-5p	hsa-miR-424-5p
none	0.31 (0.10–0.86)*	0.50 (0.19–1.27)	0.28 (0.12–0.59)*
age [years]	0.33 (0.13–0.92)*	0.49 (0.19–1.26)	0.27 (0.12–0.59)*
packyears [years]	0.31 (0.10–0.86)*	0.50 (0.19–1.27)	0.27 (0.12–0.59)*
gender	0.29 (0.09–0.82)*	0.48 (0.18–1.23)	0.28 (0.12–0.59)*
genetic risk score	0.38 (0.10–1.33)	0.53 (0.13–2.12)	0.21 (0.07–0.56)*
glaucoma	0.15 (0.04–0.54)* 1	0.23 (0.06–0.78)* 1	0.24 (0.08–0.65)*
hsa-mir-451a-5p	0.38 (0.12–1.08)	0.68 (0.24–1.85)	0.35 (0.14–0.78)*

1 strong increase in association signal by adjusting for glaucoma as a covariate.

*statistically significant association (p<0.05).

**Figure 1:**
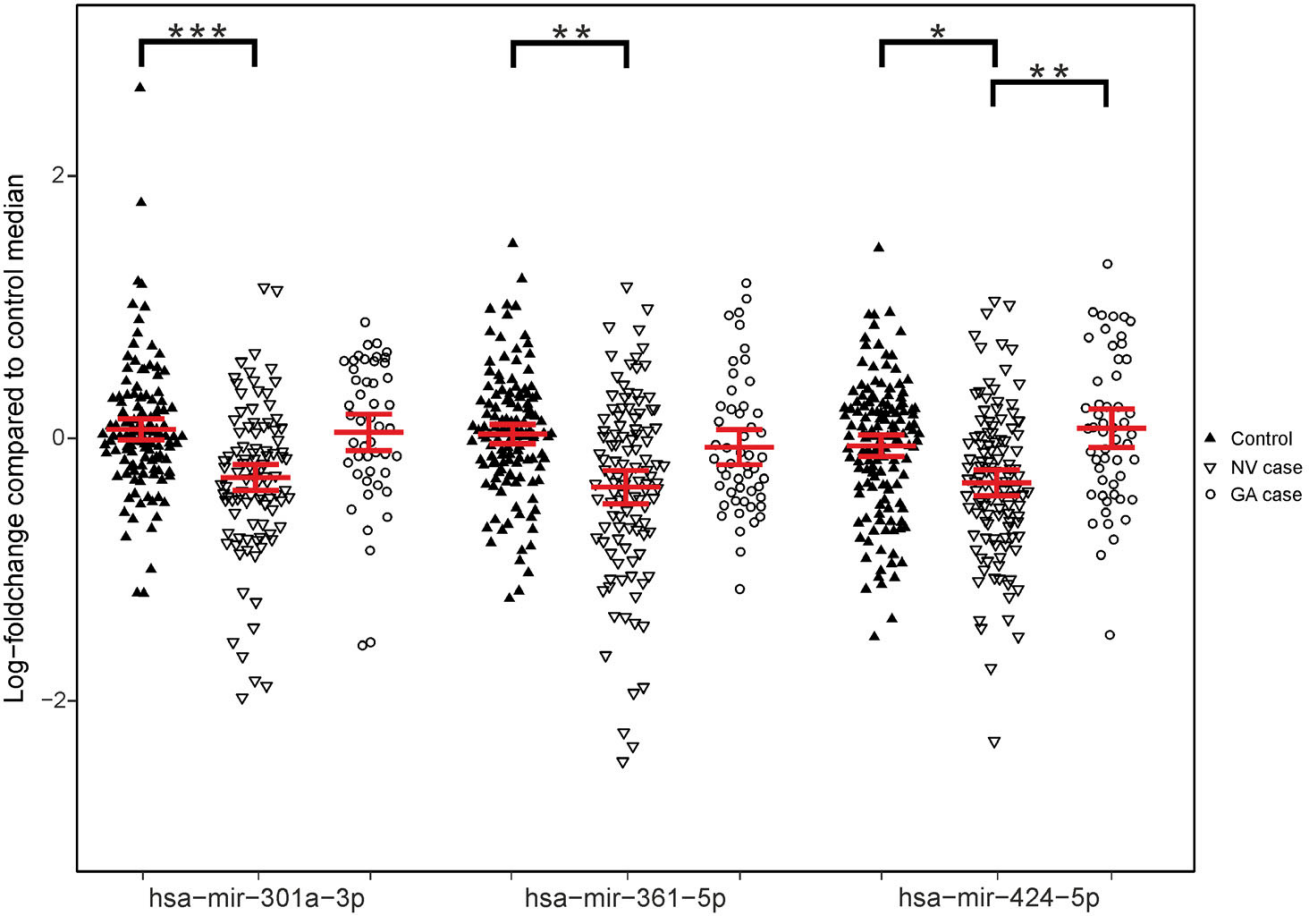
Expression analysis of three cmiRNAs (hsa-mir-301a-3p, hsa-mir-361-5p and hsa-mir-424-5p) in 129 NV AMD cases, 59 GA AMD cases and 147 healthy controls. Expression values for all samples were normalized by the median expression value in controls. Broad horizontal bars represent the mean value in each group (NV cases, GA cases or controls) for each cmiRNA. Smaller horizontal bars represent the 95% confidence intervals for each mean (see Table S1). Significant differences between means are indicated by asterix. * = p_corrected_<0.05; ** = p_corrected_<0.005; *** = p_corrected_<0.0005.

Circulating miRNAs hsa-mir-301-3p, hsa-mir-361-5p, and hsa-mir-424-5p were then analyzed by qRT-PCR in an additional replication study (Cologne study) consisting of 75 NV cases and 70 controls. In concordance with the Regensburg study, we also found reduced levels of those three cmiRNAs in NV cases compared to controls in the Cologne study. The results of the two replications were pooled and jointly analyzed (**Figure 1** **, Table S1**). We found raw (one-sided) p-values of 2.78×10^−7^, 4.09×10^−6^, and 4.75×10^−5^ for hsa-mir-301-3p, hsa-mir-361-5p, and hsa-mir-424-5p, respectively. The p-values were adjusted by a conservative Bonferroni correction, assuming 203 statistical tests based on the number of microRNAs detected in the serum of cases and controls. After correction, the p-values for hsa-mir-301-3p, hsa-mir-361-5p, and hsa-mir-424-5p were 5.63×10^−5^, 8.03×10^−4^, and 9.64×10^−3^, respectively. A cmiRNA profile including hsa-mir-301-3p, hsa-mir-361-5p, and hsa-mir-424-5p was significantly associated with AMD in the combined study (129 NV AMD versus 147 controls, p = 1.17*10^−8^) as well as in the Cologne study alone (75 NV cases and 70 controls, p = 2.43*10^−5^).

### Testing of cmiRNAs specificity in NV and GA AMD

The expression of hsa-mir-301-3p, hsa-mir-361-5p, and hsa-mir-424-5p was analyzed by qRT-PCR in the serum of 59 GA AMD patients from the Cologne and Bonn study and compared to all controls (**Figure 1** **, Table S1**). There was no statistically significant association of cmiRNA levels with GA compared to controls (p_corrected_>0.05). We also found no significant association of the cmiRNA profile including hsa-mir-301-3p, hsa-mir-361-5p, and hsa-mir-424-5p with GA AMD versus controls (p = 0.084).

Circulating miRNA hsa-mir-424-5p showed significantly higher levels in GA compared to NV (p_corrected_<0.005), while hsa-mir-301-3p and hsa-mir-361-5p were not significant (p_corrected_ >0.05).

### Pathway analysis

Pathway enrichment analysis was performed for 3,516 genes predicted by microT-CDS to be regulated by either hsa-mir-301-3p, hsa-mir-361-5p, or hsa-mir-424-5p. A total of 410 genes was predicted to be regulated by at least two of the three cmiRNAs and 35 genes were regulated by the three cmiRNAs jointly (**Figure S1**). Evaluation with miRSystem implicated the canonical TGF-β and mTOR pathways as well as related pathways such as WNT signaling, focal adhesion, neutrophin signaling and insulin metabolism as the top regulated pathways. This is in agreement with the results of mirPATH v2.0, which implicated mTOR (KEGG ID: hsa04150, p<10^−13^) and TGF-β pathways (KEGG ID: hsa04350, p<10^−14^) as top regulated pathways (**Table 4**).

**Table 4:** Pathway enrichment analysis performed with miRSystem and mirPATH2.

	genes observed/genes in pathway		genetic association reported^1^
Canonical pathway (ID^2^)	miRSystem	mirPATH2	
TGF-β signaling (hsa04350)	25/84	35/80	TGFBR1 [8]
mTOR signaling (hsa04150)	16/52	33/60	VEGFA [17]
Neutrophin signaling (hsa04722)	38/127	-	-
WNT signaling (hsa04310)	48/150	-	
Focal adhesion (hsa04510)	43/199	-	VEGFA [17]
Insulin signaling (hsa04910)	35/137	-	
Melanogenesis (hsa04916)	28/101	-	

1 genetic associations were reported in or near genes in this pathway by genome wide association studies.

^2^KEGG pathway ID (http://www.genome.jp/kegg/).

### Functional characterization of candidate miRNAs in human endothelial cells

**Figure 2:**
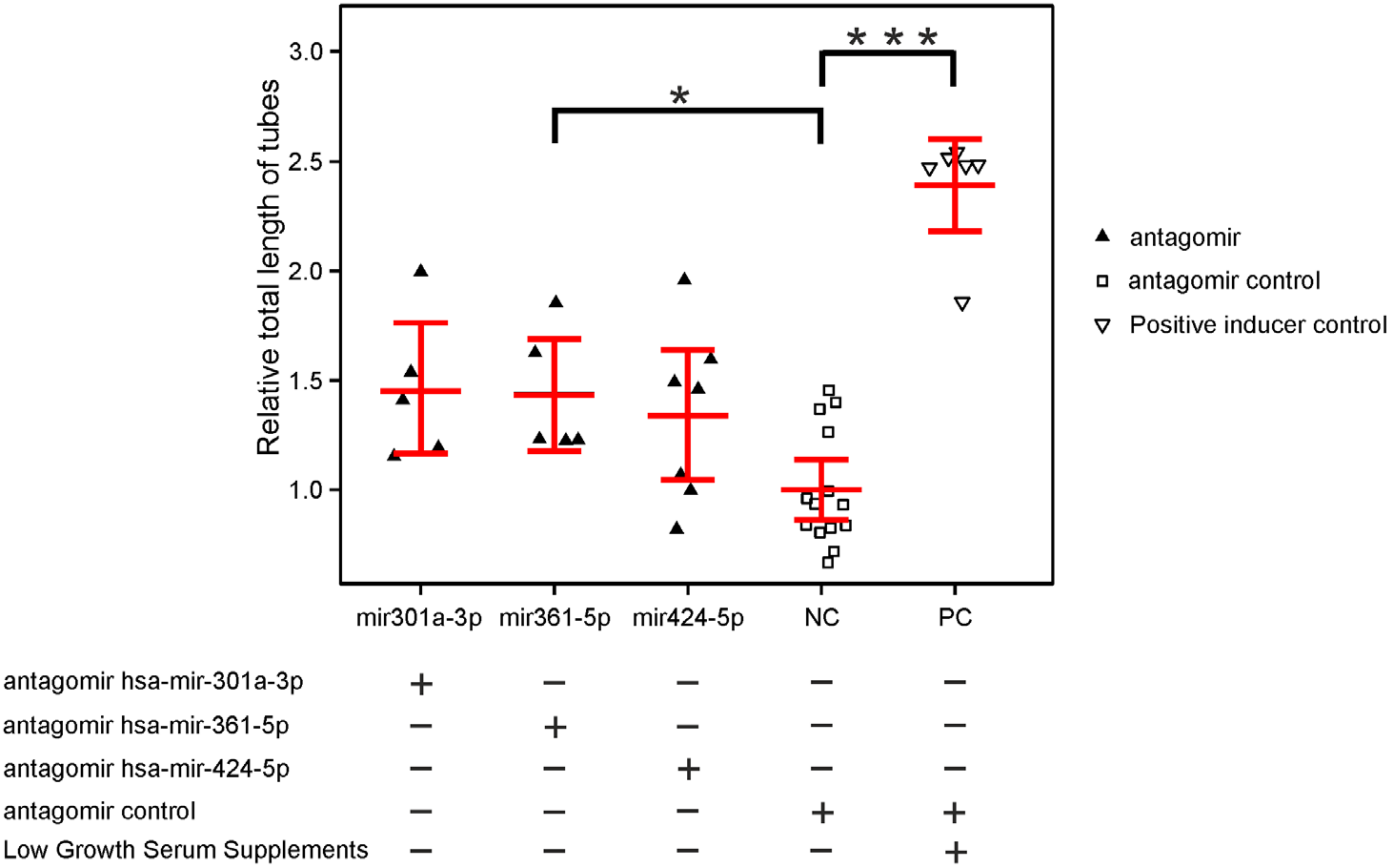
*In vitro* tube formation assays in human endothelial cells. HUVEC cells were transfected with antagomirs for hsa-mir-301a-3p, hsa-mir-361-5p or hsa-mir-424-5p or with control antagomirs (see **Figure S2**) and seeded on Geltrex/Matrigel. Cumulative tube length was quantified with Angiogenesis Analyzer implemented in ImageJ. Each measurement point indicates one independent transfection. Low Serum Growth Supplements (Life) were used as a positive inducer control. Representative images are shown in **Figure S3**. Significant differences between means are indicated by asterix. * = p_corrected_<0.05; *** = p_corrected_<0.0005.

MicroRNA hsa-mir-361-5p was shown earlier to influence the expression level of VEGFA [14] and thus should also influence angiogenesis. In order to test this hypothesis *in* *vitro*, we designed antisense oligoribonucleotides against hsa-mir-361-5p but also against hsa-mir-301a-3p and hsa-mir-424-5p and performed tube formation assays with human umbilical vein endothelial cells (HUVEC). We show that a knockdown of hsa-mir-361-5p significantly alters tube formation *in* *vitro* (p_corrected_<0.05, **Figure 2** **, Figure S2 and S3**). Knockdown of hsa-mir-301a-3p and hsa-mir-424-5p also showed elevated average tube lengths, however, this was not statistically significant after adjustment for multiple testing (p_corrected_>0.05).

### Classification

The raw AUC value for the cmiRNA profile was 0.727 for NV AMD and controls from the Regensburg study and 0.802 when restricting the analysis to NV AMD and controls from the Cologne study. Additionally, we used the weights obtained from the Regensburg study of each cmiRNA in the profile to predict the outcome (case or no case) in the Cologne study and found an AUC value of 0.722. To estimate non-parametric confidence intervals, we performed a 2,000 fold bootstrap analysis in the pooled study. The bootstrapped AUC value for the profile was 0.730 (95% CI: 0.544–0.877) indicating a good classification accuracy.

## Discussion

To our knowledge, this is the first study to evaluate the relative abundance of cmiRNAs in the serum of late stage AMD patients. We identified three cmiRNAs (hsa-mir-301a-3p, hsa-mir-361-5p, and hsa-mir-424-5p) which were significantly altered in NV AMD patients compared to AMD-free controls. Even when conditioned on covariates such as age, gender, smoking or genetic risk scores computed from known AMD-associated variants, the three cmiRNAs showed little alteration in their association strength, indicating a true association with late stage NV AMD. In contrast, there was no association of cmiRNAs hsa-mir-301a-3p, hsa-mir-361-5p, or hsa-mir-424-5p with GA AMD, suggesting subtype-specific cmiRNA profiles for late stage AMD. A global screening strategy similar to the one applied in this study may be suited to eventually characterize a GA AMD specific cmiRNA profile.

Our initial discovery study comprised 9 NV AMD cases and 9 matched controls and identified several cmiRNA candidates with altered expression levels although none reached statistical significance after adjustment for multiple testing (n = 203 equivalent to the discovery of 203 cmiRNAs). A recent study compared cmiRNA levels in long-surviving versus short-surviving patients with lung cancer and found fold changes of significantly altered cmiRNAs between 1.60 and 7.15 [15] and Cohen’s effect sizes between 0.92 and 1.54 which are considered to be large [16]. Given the number of samples in our discovery study, we calculated the power to detect comparable effect sizes after adjustment for multiple testing between 4.2% and 33.2%. This would imply a power to identify between 4 and 33 cmiRNAs out of 100 in our discovery study at the assumed effect size or higher. To compensate for lower effect sizes, we increased our sample size to 276 individuals (129 NV cases and 147 AMD-free controls) in the replication and retested individually the top 10 cmiRNAs hits from discovery. This uncovered a statistically significant association of NV AMD with cmiRNAs hsa-mir-301a-3p, hsa-mir-361-5p, and hsa-mir-424-5p.

Bioinformatical pathway analysis for genes suggested to be regulated by the NV AMD associated cmiRNAs were performed with two independent programs including the miRSystem and mirPATH v2.0. Both revealed concurring results and implicated the TGF-β and mTOR pathways in neovascular AMD pathology. Interestingly, this is in agreement with a recently published GWAS which also implicated the TGF-β and the mTOR pathways in late stage AMD by identifying risk associated genetic variants near or within the genes encoding the transforming growth factor, beta receptor 1 (*TGFBR1)* and the vascular endothelial growth factor A (*VEGFA)* [8], [17]. The TGF-β as well as the mTOR pathway are involved in cellular responses to stress and injury and also regulate angiogenesis. Consequently, we performed *in* *vitro* tube formation assays and reduced the levels of hsa-mir-424-5p, hsa-mir-301a-3p, and hsa-mir-361-5p by antisense oligoribonucleotides to evaluate the impact of decreased miRNA levels on angiogenesis. Knockdown efficiency reduced microRNA levels in the test system on average by about two-fold. Antisense treatment of hsa-mir-361-5p lead to a significant increase in tube formation and, thus, angiogenesis in vitro. Results for hsa-mir-424-5p and hsa-mir-301a-3p revealed a similar direction of effect but were not statistically significant due to correction for multiple testing. Together, the data are promising and support our bioinformatical analyses.

Additionally, pathways closely related to the mTOR pathway were implicated by our analysis including WNT signaling, focal adhesion, neutrophin signaling and the insulin pathway. These pathways are involved in (neural) cell survival and therefore are reasonable candidate pathways for the pathogenesis of AMD. However, so far no genetic association with late stage AMD was observed for any genes associated with these signaling pathways. In this context, it should be noted that until now only few studies evaluated a genetic association for progression and severity of AMD [18], [19]. These studies mainly focused on strong (and known) signals associated with increased risk for AMD and therefore may have missed possible existing associations. The present study has now identified cmiRNAs hsa-mir-301a-3p, hsa-mir-361-5p, and hsa-mir-424-5p as new biomarkers for late stage neovascular AMD. Furthermore, our data show that these biomarkers are not associated with GA AMD implying that different biomarkers and thus different biological pathways are likely involved in subtype-specific manifestations of late stage AMD. If confirmed, this could have major implications for designing treatment regiments for AMD.

A recent study investigated a treatment option for patients with stroke by increasing a disease-related reduction in plasma levels of hsa-mir-424-5p [20]. In an inducible mouse model of acute stroke which also revealed a down-regulation of hsa-mir-424-5p in plasma as well as in brain, lentiviral overexpression of hsa-mir-424-5p in the murine brain prior to induction of ischemic stroke significantly lowered the infarct volume as well as the brain edema levels [20]. A similar approach could be envisioned for treating AMD lesions. The identification of cmiRNAs that are dysregulated in NV AMD patients, now offers a number of novel starting points for therapeutic regimens. For example, such targets could be the genes that are regulated by the cmiRNAs or, alternatively, could directly address the dysregulated cmiRNAs itself. Specifically, the latter approach would initially entail prescreening of patients for altered cmiRNAs levels. Reduced expression of a diagnostic cmiRNA (as pre-microRNA or mature microRNA) could be supplemented by lentiviral transduction, nano-particle aided transfection or by delivery of the dysregulated cmiRNA via synthetic microRNAs in artificial exsosomes. Therapies to modify up- or down-regulated genes are also conceivable. This could be done by using small molecules to influence gene activity [21], protein activity and stability [22] or by targeting proteins or interacting proteins with specific antibodies [23].

In summary, this study has identified three cmiRNAs with a significantly altered expression profile in the serum of NV AMD patients when compared to AMD-free control individuals. This finding opens up a number of new avenues in understanding disease mechanisms and designing targeted treatment options. Another important aspect of our finding pertains to monitoring treatment effects in clinical trial settings. Although proof of concept is still waranted, measuring drug responses as a means of measuring changes in the cmiRNA profil from blood samples of AMD patients may proof a direct and little invasive approach in the future.

## Materials and Methods

### Ethics statement

This study followed the tenets of the declaration of Helsinki and was approved by the Ethics Review Board at the University of Regensburg, Germany (ID: 12-101-0241), University of Bonn, Germany and University of Cologne, Germany. Informed written consent was obtained from each proband after explanation of the nature and possible consequences of the study.

### Recruitment of AMD cases and control individuals

The case-control sample included 54 individuals with seemingly non-familial NV AMD and 77 age- and gender-matched AMD-free controls from the Regensburg study, 116 cases and 70 controls from the Cologne study, and 18 GA AMD cases from the Bonn Eye Clinic (**Table 1**). Inclusion and exclusion criteria have been described elsewhere [8], [24]–[26].

### Genotyping of samples

Genotyping was carried out as described elsewhere [9]. Briefly, genomic DNA was extracted from peripheral blood leukocytes. Ten single nucleotide polymorphisms (SNPs, **Table S2**) were genotyped either by direct sequencing, restriction enzyme digestion of PCR products (RFLP) or TaqMan SNP Genotyping (Applied Biosystems, Foster City, USA).

### Isolation of cmiRNAs from stabilized blood samples and serum

To reduce degradation of microRNAs and other RNA species [27], for the Regensburg and Bonn samples peripheral venous blood was drawn in PAXgene Blood RNA tubes (PreAnalytiX GmbH, Hombrechtikon, CH) and immediately stored at −80°C. To isolate RNA, tubes were thawed at room temperature on a rocker and centrifuged for 5 minutes at 1500 rcf at 4°C. The RNA isolation was carried out with the mirVANA microRNA isolation kit (Ambion, Austin, TX, USA) as described elsewhere [28]. Briefly, 300 µl of the supernatant were mixed with 600 µl of binding/lysis buffer. Then, 90µl of microRNA homogenate additive was added, thoroughly mixed for 30s and incubated on ice for 10 minutes. An equal amount of acid/phenol/chloroform (Ambion) was then added to each aliquot and vortexed for 1 minute at maximum setting. The solution was spun for 10 minutes at 10,000 g at room temperature. The resulting aqueous (upper) phase was mixed with 1.25 volumes of 100% ACS grade ethanol and passed through a mirVANA column in sequential 700 µl steps. The columns were then washed according to the manufactures protocol and the RNA was eluted with 50 µl nuclease-free water (preheated to 95°C).

For the Cologne samples, RNA isolation from blood serum was carried out with the miRNeasy Serum/Plasma kit (Qiagen) according to the manufacturer’s recommendations. Typically, we used 200 ul of serum and eluted the RNA in 24 ul of nuclease-free water.

### Sequencing of cmiRNAs and data analysis (discovery study)

cDNA libraries were constructed using the Ion Total RNA-Seq v2 kit (Life Technologies) according to the manufacturers recommendations for 9 NV AMD cases and 9 control samples. The resulting cDNA libraries were purified by AMPure beads (Beckman Coulter), and their concentrations and sizes distribution were determined on an Agilent BioAnalyzer DNA high-sensitivity Chip (Agilent Technologies). Emulsion PCR and enrichment of cDNA conjugated particles were performed with an Ion OneTouch 200 Template Kit v2 DL (Life Technologies) according to the manufacturer's instructions. The final particles were loaded on an Ion 316 chip and sequenced on a Personal Genome Machine with 200 bp read length (Life Technologies).

The data obtained were analyzed with the mirDEEP2 package [29]. Briefly, all reads were mapped to the human genome. Reads that failed to align were excluded. Remaining reads were then mapped to the pre-microRNA and microRNA sequences obtained from mirbase.org (Release 19, August 2012) and quantified. Reads per microRNA were normalized to the overall number of reads and normalized to 100,000 reads. The data were transformed with the natural logarithm to obtain a normal distribution of expression values. In order to account for batch effects in the data, we employed an empirical Bayesian batch effect correction algorithm known as ComBat [30]. For each microRNA, mean values of cases were compared to mean values of controls via t-test. Nominal significant associations with a (two-sided) p-value<0.1 were considered for replication.

### Quantitative (q)RT-PCR and data analysis (replication study)

Circulating miRNA was extracted from blood as described above and reverse transcription followed by qRT-PCR was performed according to Hurteau et al. [31]. Briefly, 10 µl of purified cmiRNA solution were modified by *E. coli* Poly (A) Polymerase I (E-PAP) by the addition of a polyA tail (Ambion, Austin, TX, USA). Reverse transcription was performed with Superscript III reverse transcriptase (Invitrogen Carlsbad, CA) and a Universal RT oligonucleotide primer, which contains a polyT stretch of DNA that binds to the newly synthesized polyA tail (**Table S3**). The RT solution was diluted 1∶50, of which 4∶µl were used per qRT-PCR reaction. Each qRT-PCR master mix was prepared according to the protocol of the Power SYBR Green Master Mix (Applied Biosystems, Foster, CA, USA) and run on an ABI Viia-7 (Applied Biosystems, Paisley, UK). Each microRNA was assayed in triplicates. Primers that performed poorly (<50% qRT-PCR efficiency) were excluded from further analysis. We further excluded measurements with a standard deviation greater than 0.4 Ct values in the triplicates. In order to normalize the Ct-values according to the amount of isolated RNA and reverse transcription efficiency, we used hsa-mir-451-5p as a housekeeping cmiRNA. This microRNA showed the least variance between cases and controls and within each group in our discovery study and was therefore regarded suitable as a housekeeper. The normalized Ct values of each individual were then normalized versus the median of the Ct values of the controls. We considered associated cmiRNAs with an odds ratio greater than 2 or lower than 0.5 for further replication.

The standard student’s t-tests was applied to evaluate a statistically significant association as implemented in R [32]. In the final dataset, we adjusted the observed raw p-values (p_uncorrected_) by a conservative Bonferroni correction (p_corrected_). Adjusted p-values below 0.05 were considered significant. Sensitivity analysis was carried out by fitting logistic regression models adjusted for possible confounding variables.

### Target prediction for cmiRNAs

We used miRSystem [33] and DIANA mirPATH v2.0 [34] to identify canonical pathways involved in AMD pathogenesis based on differentially regulated microRNAs. We used the default settings in miRSystem to identify target genes and to find canonical KEGG pathways. With mirPATH v2.0, targets predicted by microT-CDS were selected with a threshold of 0.7. The intersection of pathways which showed an involvement of all investigated microRNAs (p-value threshold: 0.005, with Conservative Stats) was considered. We excluded KEGG pathways with more than 200 genes to increase specificity and to exclude pathways considered to be too general. Furthermore, we excluded validated cmiRNA targets as well as cancer pathways such as prostate cancer (hsa05215) or glioma (hsa05214), as the majority of the cmiRNA work has been in the field of oncology and thus cancer pathways are expected by design to be among the top findings.

### Classification of cases and controls

Area under the curve (AUC) measurements were carried out with the function lroc from the package “epicalc” [35]. We used a bootstrap (n = 2000) approach to calculate robust mean and confidence interval estimates for the AUC measurements by randomly selecting half of the cases and half of the controls (with replacement) and calculating the risk model with this sub-sample (training data). A randomly selected sample of half of the cases and half of the controls (with replacement) was then used to calculated the AUC (test data).

### *In vitro* angiogenesis assay

Pooled human umbilical vein endothelial cells (HUVECs) were purchased from Life Technologies and cultured in Medium 200PRF with Low Serum Growth Supplement and Gentamicin/Amphotericin Solution (Life Technologies). Transfection of HUVECs was carried out as described in Bonauer et al. 2009 [36]. Briefly, cells were subcultured to passage 3 and grown until 70% confluent. 2′O-methyl antisense oligoribonucleotides against hsa-mir-424-5p (5′-UUCAAAACAUGAAUUGCUGCUG-3′), hsa-mir-301a-3p (5′-GCUUUGACAAUACUAUUGCACUG-3′) or hsa-mir-361-5p (5′-ACAGGCCGGGACAAGUGCAAUA-3′) or GFP (5′-AAGGCAAGCUGACCCUGAAGUU-3′) were synthesized by VBC Biotech and 50 nM were transfected with GeneTrans II (MoBiTec) according to the manufacturer’s protocol. After 24 h the medium was changed to full growth medium with supplements and antibiotics. 48 h after transfection, 3.5×10^4^ HUVECs of each transfection were sown onto one well of a 24 well plate coated with 150 µl Geltrex (Life Technologies). As a positive inducer control, cells were cultured in full growth medium with supplements. Total tube length was quantified after 24 hours by measuring the cumulative tube length in four random fields (area in each field: 2.25 mm^2^) using the Angiogenesis Analyzer in ImageJ [37]. In total, we performed between 5 and 14 independent transfections for each knockdown or control experiment. In order to assess the transfection efficiency, miRNAs were isolated with the mirVANA microRNA isolation kit (Ambion, Austin, TX, USA) according to the manufacturer’s protocol. cDNA synthesis and qRT-PCR was carried out as described above.

## Supporting Information

Figure S1 **Venn diagram of target genes predicted by microT-CDS.** Target genes were predicted with microT-CDS with a microT threshold of 0.7. In total, 3,516 target genes were predicted.
(TIF)

Figure S2 **Knockdown of candidate miRNAs in human endothelial cells.** Mean relative reduction in miRNA levels compared to control antagomir (mock). Whiskers represent the standard error of the mean.
(TIF)

Figure S3 **Representative images of** ***in*** ***vitro*** **tube formation assays in human endothelial cells.** The measured cumulative tube length in each image was close to the mean cumulative tube length measured in all images of the respective treatment.
(TIF)

Table S1 **Mean and 95% confidence intervals of log transformed fold changes of cmiRNA levels in the combined study.**
(DOCX)

Table S2 **Previously published associated variations used to calculate the genetic risk score.**
(DOCX)

Table S3 **Primers and mature microRNA sequences.**
(DOCX)
