# Supplementary figures and images for: A Circulating MicroRNA Profile Is Associated with Late-Stage Neovascular Age-Related Macular Degeneration
Source: PLoS One. 2014 Sep 9;9(9):e107461. doi: 10.1371/journal.pone.0107461 (PMC4159338; doi:10.1371/journal.pone.0107461)

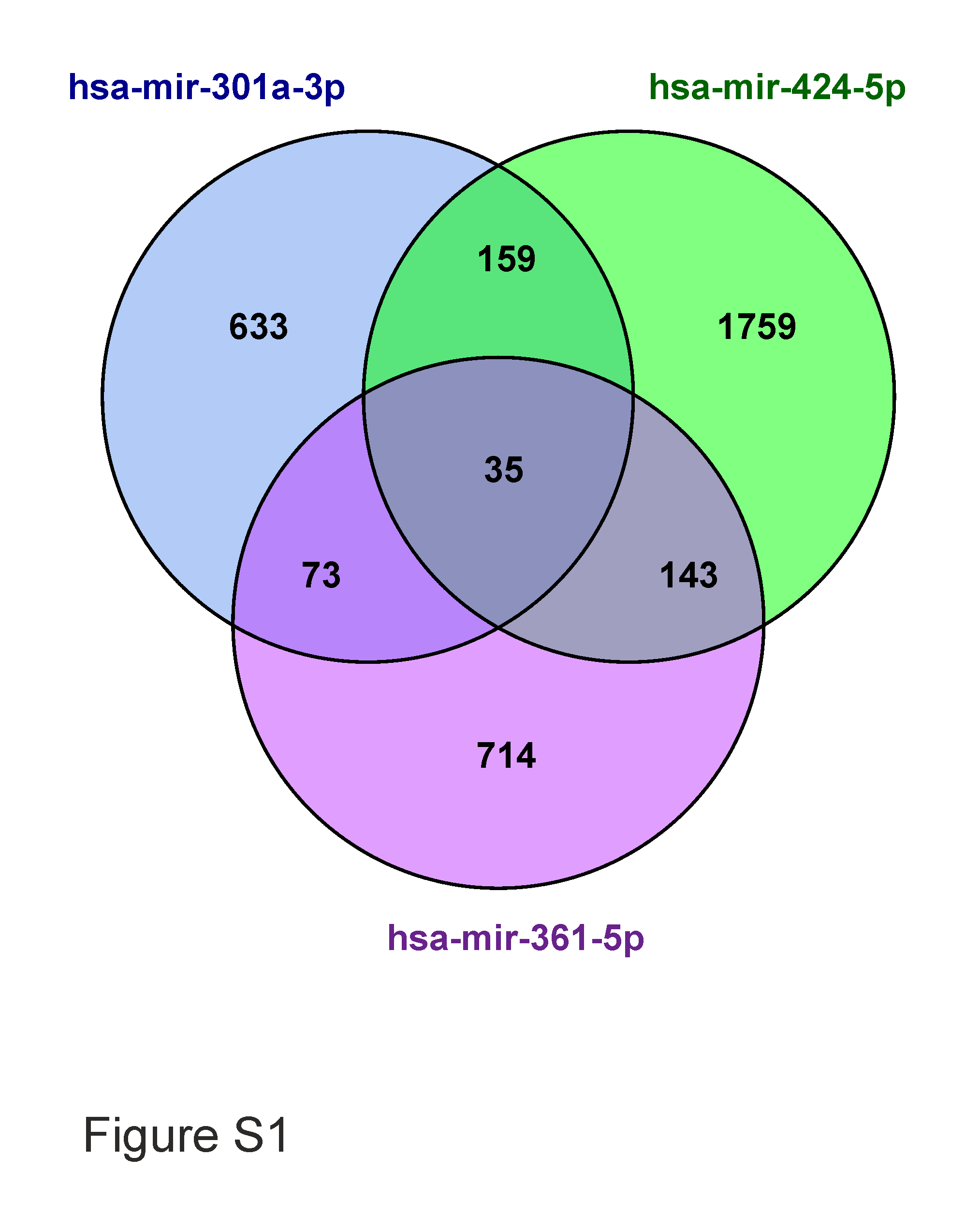

Supplement: Figure S1 — Venn diagram of target genes predicted by microT-CDS. Target genes were predicted with microT-CDS with a microT threshold of 0.7. In total, 3,516 target genes were predicted. (TIF) [file pone.0107461.s001.tif]

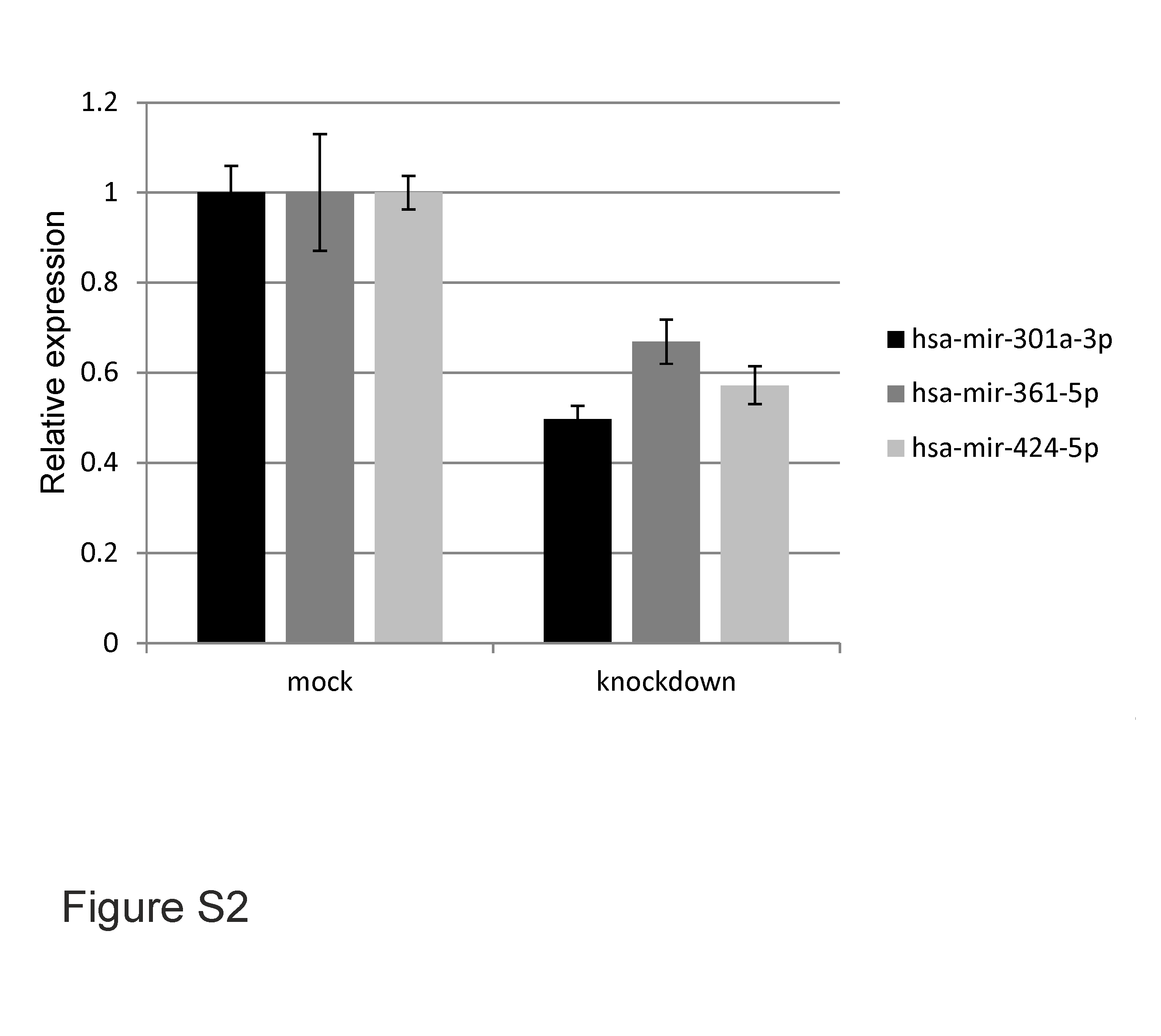

Supplement: Figure S2 — Knockdown of candidate miRNAs in human endothelial cells. Mean relative reduction in miRNA levels compared to control antagomir (mock). Whiskers represent the standard error of the mean. (TIF) [file pone.0107461.s002.tif]

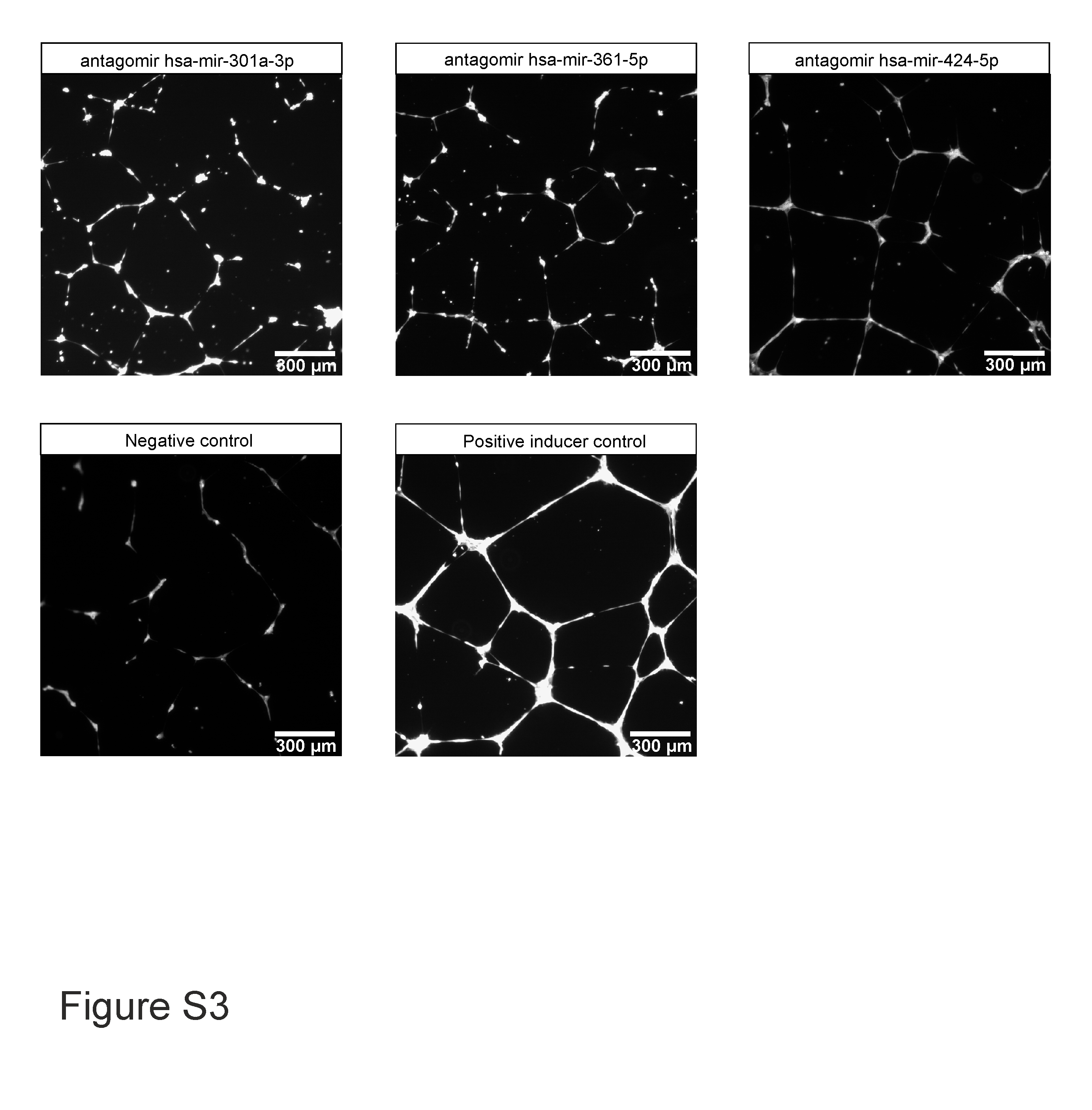

Supplement: Figure S3 — Representative images of in vitro tube formation assays in human endothelial cells. The measured cumulative tube length in each image was close to the mean cumulative tube length measured in all images of the respective treatment. (TIF) [file pone.0107461.s003.tif]
