# Supplementary material for: A Circulating MicroRNA Profile Is Associated with Late-Stage Neovascular Age-Related Macular Degeneration
Source: PLoS One. 2014 Sep 9;9(9):e107461. doi: 10.1371/journal.pone.0107461 (PMC4159338; doi:10.1371/journal.pone.0107461)
Supplement: Table S1 — Mean and 95% confidence intervals of log transformed fold changes of cmiRNA levels in the combined study. (DOCX) [file pone.0107461.s004.docx]

|  | NV cases | GA cases | controls |
| --- | --- | --- | --- |
| Number of individuals | 129 | 59 | 147 |
| hsa-mir-301a-3p | -0.318 (-0.416 - -0.220) | 0.030 (-0.110 - 0.170) | 0.055 (-0.025 - 0.134) |
| hsa-mir-361-5p | -0.373 (-0.500 - -0.245) | -0.069 (-0.203 - 0.065) | -0.002 (-0.076 - 0.072) |
| hsa-mir-424-5p | -0.338 (-0.437 - -0.240) | 0.077 (-0.068 - 0.223) | -0.071 (-0.152 - 0.011) |

**Supporting Table S1: Mean and 95% confidence intervals of log transformed fold changes of cmiRNA levels in the combined study**
