# Supplementary material for: A Circulating MicroRNA Profile Is Associated with Late-Stage Neovascular Age-Related Macular Degeneration
Source: PLoS One. 2014 Sep 9;9(9):e107461. doi: 10.1371/journal.pone.0107461 (PMC4159338; doi:10.1371/journal.pone.0107461)
Supplement: Table S2 — Previously published associated variations used to calculate the genetic risk score. (DOCX) [file pone.0107461.s005.docx]

|  |  |  |  |  |  |  |  |  | **Frequency of risk allele in** | |
| --- | --- | --- | --- | --- | --- | --- | --- | --- | --- | --- |
| **Nearby gene(s)** | **Marker** | **ID** | **Impact/effect of variant** | **Odds ratio** | **95% CI^1^** | **P-value** | **Non risk allele** | **Risk allele^2^** | **Cases (N=72)** | **Controls (N=77)** |
| *CFH* | rs1061170 | 1 | p.Y402H | 2.18 | 1.33-3.68 | 0.001762 | T | C | 0.59 | 0.36 |
|  | rs800292 | 2 | p.I62V | 1.76 | 0.79-4.06 | 0.1662 | A | G | 0.89 | 0.83 |
|  | rs6677604 | 3 | proxy for ∆CFHR3/CFHR1 | 1.96 | 0.94-4.28 | 0.0742 | A | G | 0.87 | 0.79 |
| *ARMS2* | rs10490924 | 4 | p.A69S | 5.62 | 2.90-12.01 | 2.63E-08 | G | T | 0.55 | 0.19 |
| *CFB* | rs4151667 | 5 | p.L9H | 8.47 | 1.48-160.00 | 0.01319 | A | T | 0.99 | 0.93 |
|  | rs438999 | 6 | proxy for rs641153 (p.R32Q) | 2.29 | 0.77-7.73 | 0.1374 | C | T | 0.95 | 0.91 |
| *C3* | rs2230199 | 7 | p.R102G | 1.73 | 0.95-3.25 | 0.07395 | G | C | 0.28 | 0.18 |
| *APOE* | rs7412 | 8 | p.R158C | 0.70 | 0.30-1.57 | 0.3924 | C | T | 0.08 | 0.12 |
|  | rs429358 | 9 | p.C112R | 1.80 | 0.76-4.54 | 0.1829 | C | T | 0.92 | 0.86 |
| *PLA2G12A* | rs2285714 | 10 | synonymous exonic, unknown | 1.19 | 0.72-1.99 | 0.492 | C | T | 0.45 | 0.40 |

**Supporting Table S2: previously published associated variations used to calculate the genetic risk score**

^1^ 95% confidence intervals

^2^ Risk allele refers to AMD risk increasing allele
