# Supplementary material for: A Circulating MicroRNA Profile Is Associated with Late-Stage Neovascular Age-Related Macular Degeneration
Source: PLoS One. 2014 Sep 9;9(9):e107461. doi: 10.1371/journal.pone.0107461 (PMC4159338; doi:10.1371/journal.pone.0107461)
Supplement: Table S3 — Primers and mature microRNA sequences. (DOCX) [file pone.0107461.s006.docx]

**Supporting Table S3: Oligonucleotide primers and mature miRNA sequences**

| **primer name** | **mature miRNA sequence** | **primer sequence** |
| --- | --- | --- |
| hsa-miR-142-RT | CAUAAAGUAGAAAGCACUACU | CATAAAGTAGAAAGCACTACT |
| hsa-miR-361-RT | UUAUCAGAAUCUCCAGGGGUAC | TTATCAGAATCTCCAGGGGTA |
| hsa-miR-424-RT | CAGCAGCAAUUCAUGUUUUGAA | CAGCAGCAATTCATGTTTTGAA |
| hsa-miR-4732-5p_RT | UGUAGAGCAGGGAGCAGGAAGCU | TGTAGAGCAGGGAGCAGGAAGCT |
| hsa-miR-451a_RT | AAACCGUUACCAUUACUGAGUU | AAACCGTTACCATTACTGAGTT |
| hsa-miR-192-RT | CUGACCUAUGAAUUGACAGCC | CTGACCTATGAATTGACAGCC |
| hsa-miR-26a-RT | UUCAAGUAAUCCAGGAUAGGCU | TTCAAGTAATCCAGGATAGGCT |
| hsa-miR-505-RT | GGGAGCCAGGAAGUAUUGAUGU | GGGAGCCAGGAAGTATTGAT |
| hsa-miR-335-5p_RT | UCAAGAGCAAUAACGAAAAAUGU | TCAAGAGCAATAACGAAAAATGT |
| hsa-miR-301a-3p_RT | CAGUGCAAUAGUAUUGUCAAAGC | CAGTGCAATAGTATTGTCAAAGC |
| hsa-miR-30b-5p_RT | UGUAAACAUCCUACACUCAGCU | TGTAAACATCCTACACTCAGCT |
| hsa-miR-194-5p_RT | UGUAACAGCAACUCCAUGUGGA | TGTAACAGCAACTCCATGTGGA |
| hsa-miR-4732-5p_RT | UGUAGAGCAGGGAGCAGGAAGCU | TGTAGAGCAGGGAGCAGGAAGCT |
| Univeral_PCR_Primer | - | AACGAGACGACGACAGACTTT |
| URT_Primer | - | AACGAGACGACGACAGACTTTTTTTTTTTTTTTV |
